# Prehospital ultrasound constitutes a potential distraction from the observation of critically ill patients: a prospective simulation study

**DOI:** 10.1186/s13049-024-01280-4

**Published:** 2024-11-10

**Authors:** Yael van der Geest, Luca Marengo, Roland Albrecht, Philipp K. Buehler, Pedro D. Wendel-Garcia, Daniel A. Hofmaenner, Urs Pietsch

**Affiliations:** 1 School of Medicine, University of Bern, Bern, Switzerland; 2 Swiss Air-Ambulance Rega, Zurich, Switzerland; 3 Institute of Intensive Care Medicine, Cantonal Hospital Winterthur, Zurich, Switzerland; 4 Institute of Intensive Care Medicine, University Hospital Zurich, Zurich, Switzerland; 5 Department of Emergency Medicine, Inselspital, Bern University Hospital, University of Bern, Bern, Switzerland; 6 Division of Perioperative Intensive Care Medicine, Cantonal Hospital St. Gallen, Rorschacher Strasse 95, 9007 St. Gallen, Switzerland

**Keywords:** Prehospital ultrasound, HEMS, Eye tracking, Situational awareness, Gaze behaviour, Human factor

## Abstract

**Background:**

Prehospital point-of-care ultrasound allows an unstable patient to be rapidly and accurately assessed. However, we are concerned that an excessive focus on the ultrasound device, in an already demanding emergency medical service environment, may distract from patient care, potentially leading to reduced situational awareness and the neglect of other crucial instruments, such as the patient monitor. Thus, in this study, we examined the influence of prehospital ultrasound on situational awareness, by studying the degree to which physicians were distracted from the patient monitor.

**Methods:**

We observed HEMS physicians in a simulated setting and analysed their gaze behaviour using an eye tracker placed on three areas of interests: the ultrasound device, the patient and the patient monitor. In the course of the experiment, the simulated patient desaturated, which was presented on the patient monitor. The primary outcome was the fraction of gaze distribution across the three areas of interest, while secondary outcomes were different gaze metrics (dwell time, revisits, average duration of visual intake and entry time) on the patient monitor. We then compared the participants who noticed the patient’s deterioration with those who did not.

**Results:**

In 75% of cases, the severely decreased oxygen saturation went unnoticed during the test. Moreover, the gaze distribution of the two groups differed, with the group that recognised the deterioration focusing longer on the patient monitor (7.8% (95% CI 5–10.7) vs 0.1% (95% CI 0–0.3), *p*: 0.124).

**Conclusions:**

The task of performing an ultrasound examination appears to overwhelm some participants and distract them from other aspects of the scenario. Efforts to mitigate distractions and optimise the use of prehospital ultrasound, such as education, a focus on human factors aspects and standardisation, are crucial for maximising the potential benefits of prehospital ultrasound.

## Background

Prehospital point-of-care ultrasound (PoCUS) has proven to be a valuable tool that enables the rapid and accurate assessment of patients in a prehospital setting and whose benefits are well documented [1, 2]. It has been shown to expedite patient management, increase diagnostic accuracy and support appropriate treatment decisions on-scene. As early as 2011, it was declared one of the five most important research priorities in physician-provided prehospital critical care [2]. In recent years, the focus has increasingly shifted from merely introducing it to training and education [3–5].

If ultrasound examinations are to improve patient outcomes, a balance must be struck between time and benefit. Moreover, it is imperative to assess which patients will benefit from examinations that may increase on-scene time and which will not.

Despite the advantages it brings, we are concerned that focusing on the ultrasound device in an already demanding environment may distract the physician’s attention from the surroundings. Various factors make examinations in an HEMS setting particularly challenging: weather conditions, light changes [3], vibrations, noise, limited medical staff resources and the unusual spatial setting.

At present, there is insufficient literature on the effects of the introduction of technical devices on human factors, such as situational awareness.

In order to measure situational awareness and distraction, we used an eye tracker. Eye-tracking technology has proven to be a suitable instrument in various studies, as it objectifies gaze behaviour and thus provides insight into participants’ areas of interest during a complex exercise [6–8]. By means of infrared light that reflects off the cornea and is captured by cameras, the eye tracker can catch eye movements and thereby assess visual behaviour. This objective form of observation is superior to conventional ones, where expectation bias cannot be prevented [6]. Since visual behaviour can be linked to cognitive processing in physicians, the eye tracker represents a valuable tool for assessing situational awareness [9].

We are concerned that an excessive focus on ultrasound examinations combined with the aforementioned challenges in an HEMS context leads to reduced situational awareness and the corresponding risk of neglecting other crucial points, such as the patient or the patient monitor. Thus, in this study, we examined the impact of prehospital ultrasound on situational awareness by studying distraction with regard to the patient monitor in a simulated setting.

## Methods

### Study design and study population

This was a prospective simulation study with HEMS physicians as participants. They were recruited on a voluntary basis during a training day at our training and simulation centre (Rea2000, St. Gallen, Switzerland) and were included if accurate technical calibration of the eye tracker was possible. In this paper, we present the results of the observational study in accordance with the STROBE guidelines [10]. Our study conforms with the ethical standards of the institutional and national research committee, as well as with the 1964 Declaration of Helsinki and its later amendments or comparable ethical standards. It was approved by the Ethics Committee of Eastern Switzerland (EKOS BASEC Nr. Req-2023-01130 EKOS 23/174).

### Simulation

We observed HEMS physicians performing an ultrasound examination on a live patient in a simulated prehospital HEMS transport setting. The simulation room consisted of a small chamber (with a helicopter-cabin-style patient monitor and seating arrangement) including a rescue stretcher, an iPad with an ultrasound probe and display application (Butterfly iQ; USA) and a patient monitor. The vital parameters displayed on the patient monitor could be modified by the observer using an iPad (iSimulate; Albany, NY, USA).

The participants were instructed that their patient was a motorcyclist who had been involved in a traffic accident (motorcyclist vs car) and that the patient had already been monitored in the helicopter. At the beginning of the examination, the patient was hemodynamically stable and only complained about a pain in the left hemithorax.

The participant was instructed to perform an ultrasound examination during transport to the nearest hospital and was asked to report all subjectively perceived pathological findings to the observer present in the room (any changes to the patient, including pathological findings observed sonographically and on the patient monitor). There was no time limit for the task, although the participant was told that it would take about five minutes. However, the participant was not interrupted by the examiner and was asked to communicate once they had finished the ultrasound examination.

Within two minutes of the session beginning, the patient desaturated (a drop in simulated oxygen saturation from 100 to 88%), as displayed on the patient monitor. We noted the time until the drop in saturation was recognised for inclusion in our statistical analysis. As in a flight scenario, the patient monitor did not emit an audible alarm to attract the participant’s attention.

### Data collection

We collected data on gaze metrics using an SMI Eye Tracking Glasses 2 Wireless system and analysed the raw data with the software SMI Be-Gaze 3.7 (Sensomotoric Instruments, Teltow, Germany).

Data on professional and sonographic experience were collected by means of a questionnaire.

The survey also included an evaluation of stress levels during the simulation, as well as any distractions or restrictions caused by the eye tracker.

The participants reported directly to the observer present in the simulation room any pathological findings such as the patient’s desaturation.

### Data analysis

On the level of gaze behavior, three specific areas of interest (AOI) were defined in advance for the purposes of statistical analysis: the ultrasound device, the patient and the patient monitor. Irrelevant gaze fixations (e.g. fixation on the floor, surroundings, etc.) were excluded from the analysis. Assuming an absolute dwell time of 1000 ms (± 400 ms) or a relative dwell time of 8 ms (± 3 ms), and with the aim of identifying a decrease in dwell time of 90% with an alpha of 0.05 and a beta of 0.1, six patients with a 1:2 distribution between groups are required.

### Statistical analysis

The data were summarised by means of median [interquartile range] and counts with percentages, as appropriate. Group level differences were assessed by means of Mann–Whitney–Wilcoxon test. Continuous visual data were analysed via linear effects models, taking visual data as the dependent variable and the non-identification of the underlying pathology as a fixed effect. Analyses were also performed across AOIs via linear mixed-effects models, taking the individual AOIs as random effects.

The statistical analysis was performed via a fully scripted data management pathway using the R environment for statistical computing version 4.3.1. A two-sided *p* < 0.05 is considered statistically significant.

### Outcome

For the primary outcome, i.e. the fraction of gaze distribution associated with the different AOIs, we compared the gaze behaviour between those participants who noticed the patient’s desaturation (red group) and those who did not (blue group). We examined gaze distribution across AOIs (the ultrasound device, the patient and the patient monitor), revisits (number of re-fixations to an AOI), entry time (time to first fixation of an AOI), average visual exposure time (average time spent on an AOI across all fixations) and dwell time (cumulative time spent on an AOI, including blinks and saccades).

## Results

### Participants

A total of eight HEMS physicians were included in the study. Data concerning their baseline characteristics, which were collected by means of a survey, are presented in Table 1.

**Table 1:** Baseline characteristics of the participants

Female sex	3 (37.5%)
*Qualification*	
Board certified anesthesia	5 (62.5%)
Board certified anesthesia + critical care	3 (37.5%)
*Education in ultrasound examinations*	
No education	1 (12.5%)
Course in POCUS	6 (75%)
Other	1 (12.5%)
Practical experience in prehospital ultrasound	1 (12.5%)
*Preclinical experience (months)*	
Ground-bound	70.13 (95% CI 11.74–128.51)
Airborne	55.25 (95% CI 9.95–100.55)
General clinical experience (years)	11.44 (95% CI 7.76–15.12)
Number of air-rescue missions (per year)	134.29 (95% CI 15.62–252.95)

According to the questionnaire, stress levels during the simulation were low (1 [IQR 0–3]), while the eye tracker only slightly impaired mobility (2 [IQR 0.5–3.5] and work performance [3.75 IQR 2–5.5]). No critical incidents occurred during the measurements.

Seven of the participants (87.5%) considered the portable ultrasound device on a helicopter to be useful, and all eight participants (100%) wanted to learn more about prehospital ultrasound.

Two participants (25%) noted the desaturation as an abnormality during the examination and asked the observer to provide the patient with oxygen, while six (75%) of the participants missed the patient’s desaturation during the examination. Furthermore, four participants (50%) felt confident with the prehospital ultrasound, including the two participants (100%) who recognised the desaturation and two out of the six (33%) who did not.

### Main analysis

**Fig. 1:**
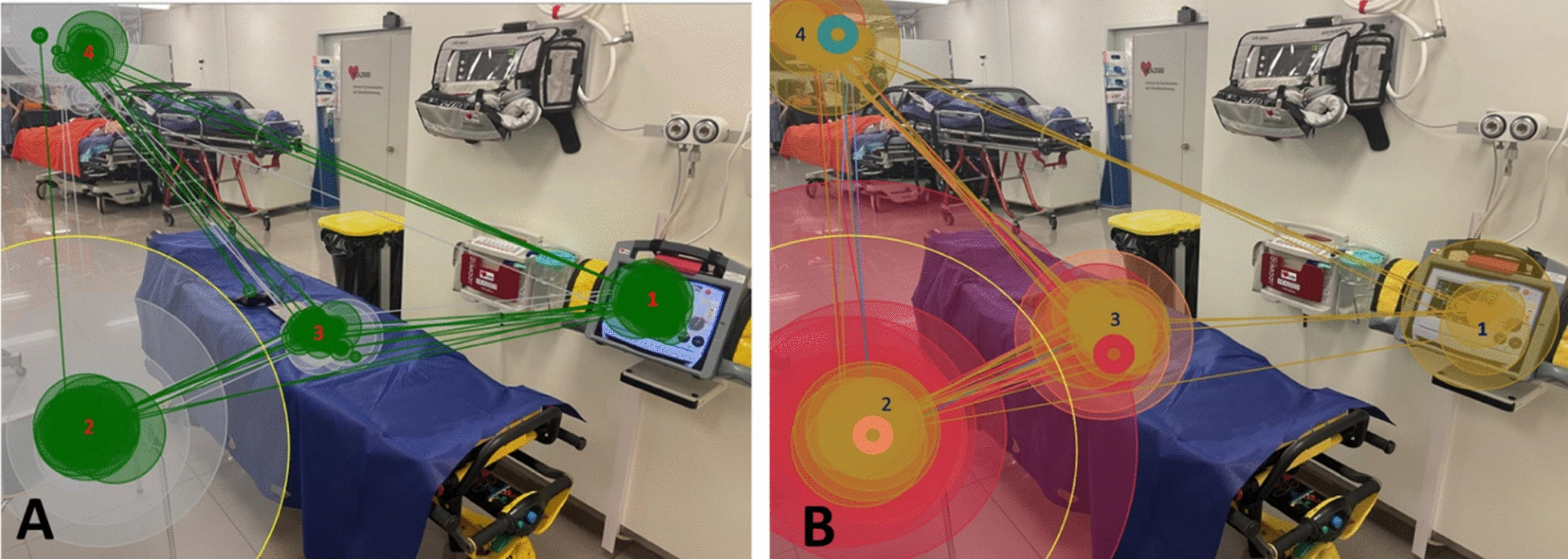
Heatmap of overall gaze behavior. Areas of interest: 1 = patient monitor, 2 = ultrasound screen, 3 = patient, 4 = blank (irrelevant fixations, e.g. on the floor). **A** Heatmap of ‘red’ group (i.e. participants who recognised desaturation); **B** Heatmap of ‘blue’ group (i.e. participants who did not recognise desaturation)

We analysed the gaze metrics (average visual intake, dwell time, revisits and entry time), taking the patient monitor as an area of interest, and divided the participants into two groups: the red group (two participants), which recognised the saturation drop, and the blue group (six participants), which did not (Fig. 1).

With regard to the overall gaze behavior, the heat maps (the visual quantification of focus on the different AOIs) reveal that the two groups have a different gaze distribution, especially in relation to the patient monitor: during the examination, the red group focused 7.8% of the time on the patient monitor, as opposed to 0.1% for the blue group.

**Fig. 2:**
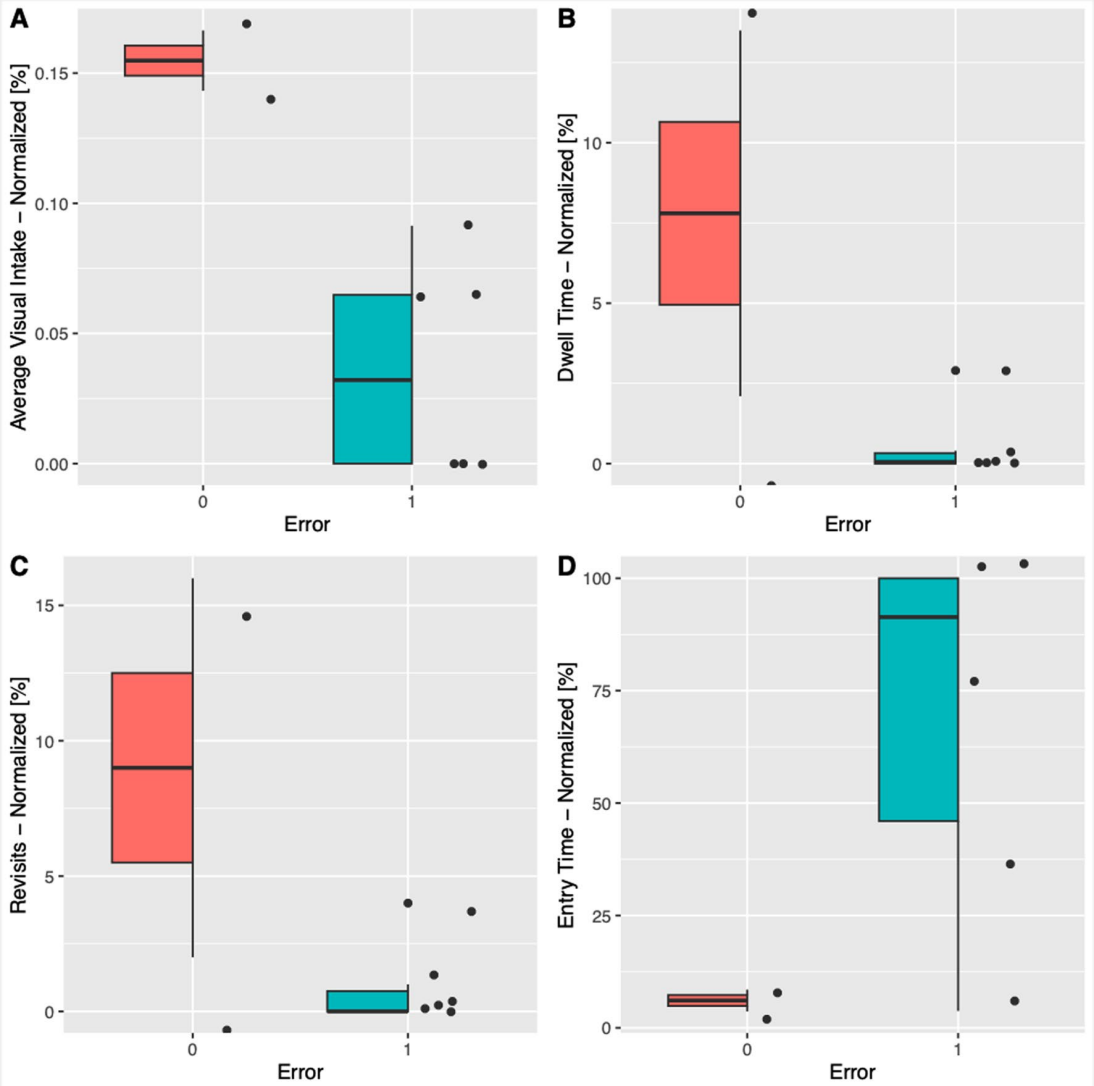
Gaze metrics for the patient monitor as an area of interest. Red bar: group of participants who noticed oxygen desaturation, blue bar: group of participants who did not notice oxygen desaturation

The box plots (Fig. 2) illustrate the different gaze parameters of the two groups, taking the patient monitor as an area of interest: the red group spent more time focusing on the patient monitor during the experiment (average visual intake 0.16% vs 0.04% (*p*: 0.009)) and remained focused on it for longer (dwell time 7.8% vs 0.6% (*p*: 0.043)). They also looked at it more frequently (revisits 18.8% vs 1.5% *p*: 0.01) and focused on it earlier (entry time 6.1% vs 70% (*p*:0.084)).

## Discussion

In this prospective simulation trial, 75% of HEMS physicians failed to notice a deterioration in their patient’s condition while performing a prehospital ultrasound examination. Moreover, their gaze distribution was different from those who recognised the deterioration, since they were more occupied with generating the ultrasound images and had significantly less time for observations besides the ultrasound device. Only 33% of the participants who missed desaturation felt confident performing the prehospital ultrasound, compared to 100% of participants who recognised the desaturation.

However, this study is subject to several limitations, including the fact that the study population consisted of a small number of participants. To address this, we carried out a statistical analysis beforehand to avoid the risk of a beta error. The participants were divided into two groups for statistical analysis after the simulation, according to whether they recognised the desaturation or not. However, the demographic characteristics, and especially data on individual ultrasound experience, were not taken into account in the statistical analysis. Further studies are necessary to examine the grade of distraction that occurs when ultrasound examinations are performed by two groups with different levels of ultrasound skills, since we expect personal experience to have an impact on performance. Furthermore, the behaviour of the participants may have been influenced by the scenario design, as the simulated patient is expected to be healthy with no pathological findings or changes to their vital parameters. The participants are clearly instructed to perform a prehospital ultrasound examination and to pay attention to any changes.

As no comparable studies exist to date, these results will serve as a basis for additional larger-scale studies. Such further studies are necessary in order to assess distractability in-depth, while taking the ultrasound experience of the study population into account.

In our simulation study, the task of performing an ultrasound examination seems to have overwhelmed some of the participants and distracted them from the other aspects of the scenario.

Indeed, during a critical emergency, it is common to be confronted with more information and tasks than one can process, leading to cognitive overload which impairs decision-making and can cause EMS providers to lose sight of the ‘big picture’. As a result, they can become fixated on a particular task, such as ultrasound or intubation [5]. 40% of reported complications to the 4th National Audit Project of the Royal College of Anaesthetists and Difficult Airway Society (NAP4) are related to ‘human factors and considered to have contributed to adverse outcomes [11]. Human factors, such as situational awareness, play a pivotal role in the effectiveness and safety of prehospital ultrasound examinations. For this reason, healthcare providers must be equipped with the knowledge and skills necessary to perform ultrasound examinations accurately, especially in difficult and distracting environments. Ongoing training and education are thus essential to improving their ultrasound expertise and navigating the sometimes chaotic prehospital setting. A focus on human factors also means that standardised protocols, training and guidelines are needed that are specifically tailored to prehospital ultrasound. In this way, healthcare providers can ensure a systematic approach to ultrasound examinations, even in high-pressure situations.

Incorporating principles related to human factors into prehospital ultrasound training programmes and protocols can significantly enhance the ability of healthcare providers to focus on conducting ultrasound examinations amid distractions, thus maximising the potential benefits of prehospital ultrasound with regard to improving emergency care.

Efforts to mitigate distractions and optimise the use of prehospital ultrasound are crucial. Strategies such as ongoing training and simulation exercises for EMS providers can help improve their ability to focus on conducting ultrasound examinations amid distractions. Furthermore, streamlining protocols and incorporating checklists specific to prehospital ultrasounds can help maintain a structured approach even in chaotic environments [11].

As mentioned above, the accuracy of prehospital ultrasound can vary depending on the experience and skill of the examiner, underlining the importance of training and education for the successful implementation of prehospital ultrasounds in emergency care.

In this context, eye tracking is a particularly helpful tool for objectively assessing the participants’ visual attention. A conventional observational approach would have been unable to establish detailed gaze distributions and could have resulted in various biases (e.g. incorrect subjective estimates of participants’ gaze metrics on the AOIs including the patient monitor). However, this tool has been applied in only a handful of studies, meaning that more research is needed to optimise the use of this resource in observational studies.

## Conclusions

The task of performing an ultrasound examination appears to overwhelm some participants and distract them from other aspects of the scenario. Efforts to mitigate distractions and optimise the use of prehospital ultrasound, such as education, a focus on human factors and standardisation, are therefore crucial for maximizing the potential benefits of prehospital ultrasound.

## Data Availability

The datasets used and/or analysed in the current study are available from the corresponding author on reasonable request.

## Abbreviations

AOI: Areas of interest
EMS: Emergency medical services
HEMS: Helicopter emergency medical services
PoCUS: Prehospital point-of-care ultrasound
